# Virtual Machine Resource Allocation Optimization in Cloud Computing Based on Multiobjective Genetic Algorithm

**DOI:** 10.1155/2022/7873131

**Published:** 2022-03-10

**Authors:** Feng Shi, Jingna Lin

**Affiliations:** ^1^Department of Computer Science and Technology, Taiyuan University, Taiyuan 030032, China; ^2^Shanxi Vocational University of Engineering Science and Technology, Taiyuan 030031, China

## Abstract

Cloud computing is an important milestone in the development of distributed computing as a commercial implementation, and it has good prospects. Infrastructure as a service (IaaS) is an important service mode in cloud computing. It combines massive resources scattered in different spaces into a unified resource pool by means of virtualization technology, facilitating the unified management and use of resources. In IaaS mode, all resources are provided in the form of virtual machines (VM). To achieve efficient resource utilization, reduce users' costs, and save users' computing time, VM allocation must be optimized. This paper proposes a new multiobjective optimization method of dynamic resource allocation for multivirtual machine distribution stability. Combining the current state and future predicted data of each application load, the cost of virtual machine relocation and the stability of new virtual machine placement state are considered comprehensively. A multiobjective optimization genetic algorithm (MOGANS) was designed to solve the problem. The simulation results show that compared with the genetic algorithm (GA-NN) for energy saving and multivirtual machine redistribution overhead, the virtual machine distribution method obtained by MOGANS has a longer stability time. Aiming at this shortage, this paper proposes a multiobjective optimization dynamic resource allocation method (MOGA-C) based on MOEA/D for virtual machine distribution. It is illustrated by experimental simulation that moGA-D can converge faster and obtain similar multiobjective optimization results at the same calculation scale.

## 1. Introduction

Cloud computing is an emerging technology in the field of computers. Although it has been applied, its development is not perfect. Many technical standards have not yet formed, and are still in the period of growth. As long as we grasp the rare opportunity, we can occupy a place in the future cloud computing field [1–3]. In this critical period of the formation and development of new technology, whoever holds the commanding heights of technology will hold the initiative of future technology strategy [4]. In addition to commercial applications, many excellent characteristics of cloud computing make it an essential aspect in military education, meteorology, astronomy, and other fields. Therefore, the development of cloud computing technology has been included in the national science and technology strategy of many countries [5–7].

**Figure 1:**
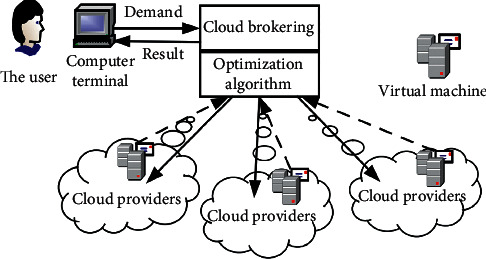
VM resource allocation in cloud computing.

In cloud computing applications, Infrastructure as a Service (IaaS) is an important service model, through which cloud providers can provide massive computing resources to cloud users through the Internet. In addition, the price fluctuation and the uncertainty of demand and other factors make this problem more complicated. Therefore, in order to meet the resource usage requirements and minimize user expenses, the VM allocation scheme must be optimized. This problem is called VM allocation optimization (shown as Figure 1) [8, 9].

**Figure 2:**
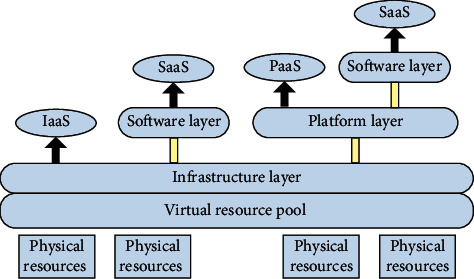
Framework diagram of the cloud computing service model.

The emergence of cloud computing platform virtualization technology brings new opportunities to realize energy saving in the cloud computing environment. The use of virtualization technology enables multiple users to share the computing resources pool according to the actual demand. The emergence of this technology has greatly improved the resources utilization. With the emergence of a shared resource pool, the number of servers in the cloud environment has reduced, thereby increasing the income of the cloud service provider [2, 10, 11]. And, resource allocation problems ultimately boils down to multiple application mapping relations between the virtual machine and the server, through reasonable allocation, adjusting the distribution of application virtual machines on the physical nodes, making full use of the service condition of the server idle resources, so as to reduce the amount of activation server, and thereby achieve the purpose of reducing energy consumption [12]. At present, there are three basic service modes in the application of cloud computing technology: Software as a Service (SaaS), Platform as a service (PaaS), and Infrastructure as a service (IaaS), as shown in Figure 2. Each service mode corresponds to certain system levels.

At present, many energy-saving resource scheduling methods of cloud platform focus on how to reduce the number of active servers to achieve energy saving and green computing. However, the above resource scheduling schemes often ignore the dynamic change of application load caused by the change of user demand in the cloud environment, and they also fail to consider the stability of application load distribution on each physical node. Although the distribution of application VMS among physical nodes in the current state makes fewer physical nodes active, the change of user requirements will lead to changes in the load of each application; in this case, the dynamic change of the required load will lead to resource hot spots on these physical nodes in the future with the change of the application load. The status of these physical nodes may reappear in the near future, which will directly trigger a new round of dynamic resource configuration requirements [13]. To solve the above problems and to put forward a new thinking of physical nodes on the stability of the energy-saving resource scheduling method, how to find a virtual machine distribution that activates the stable state of each physical node and how to reduce the energy consumption of virtual machines from the old state to the new state are urgent and necessary [14].

## 2. Related Works

With the development of cloud computing, people find that although cloud computing brings convenience, its energy consumption is also huge compared with the traditional service mode. Now, some suppliers in order to meet user requirements, which can respond to dynamic changes in user needs. However, in the actual environment, this will cause a great waste of resources and increase unnecessary energy consumption. With the change of user demand, the application load request resources will change accordingly. Therefore, how to reduce energy consumption while ensuring service quality is a problem to be solved. In a cloud computing environment, the optimal VM resource distribution scheme can effectively save energy by properly deploying VM resources on physical nodes [15, 16]. Virtual machine deployment strategy is mainly considering the distribution and use of computing resources in data center, so as to select the best distribution scheme, according to the distribution plan to make sure the virtual machine and the mapping relations between physical nodes. If the new deployment scheme satisfies the constraints of the collection, then optimal allocation can be achieved. However, the allocation of multiple virtual machines to multiple physical nodes proved to be a problem, namely NP problem. Therefore, which strategy is adopted to realize the placement of virtual machines to achieve the purpose of energy saving has always been a hot research topic in the field of cloud computing [17, 18].

At present, the research of cloud computing is still in the initial stage. In terms of the development direction of the future mainstream cloud computing industry, there are also two different camps among the big companies that master the core technology of cloud computing. Emerging companies represented by Google and Amazon are pushing the Internet cloud first, while traditional IT enterprises represented by IBM and Microsoft are pushing the IT cloud. In addition, due to commercial interests, many high-end cloud computing technologies are not disclosed as business secrets of enterprises [19]. Google is recognized as the leader in cloud computing technology, occupying a great technological advantage, but many of its technologies are not disclosed. At present, scholars mainly study cloud computing in two ways: one is based on part of the macroscopic technical data disclosed by Google and combined with traditional parallel computing-based grid computing virtualization technology [20]. The other is based on the open source project Hadoop for communication and application of different ideas and different application environments, resulting in the development of many different branches of technology. Therefore, related research on cloud computing is still in a stage of contention, and the technical system has not yet been formed.

As mentioned above, the widespread interest in cloud computing stems from its successful commercial application, so the earliest cloud computing research institutes are mainly concentrated in several major Western IT technology companies. However, with the gradual increase of the influence of cloud computing, its many excellent characteristics make people see its application in other fields such as military scientific research. Cloud computing technology began to receive attention from various countries and has become an important aspect for countries to compete for the commanding heights of future science and technology [2]. In China, in recent years, due to the strong support of the state, as well as the extensive promotion of business plans, cloud computing has gradually become a popular research content, has achieved rapid development, and its influence is also increasing. In 2010, Beijing successfully hosted the world cloud computing conference. Lenovo, Sohu, Baidu, and other domestic companies have also begun to develop business based on cloud computing. In addition, various domestic universities began to set up the relevant research direction and discipline curriculum. Cloud computing research revealed a strong momentum, and has great development potential. However, there is still a big gap between China and western countries, and more investment and policy planning are needed [21].

Resource management and allocation is a key problem in cloud computing. In the IaaS mode, resource allocation is a virtual machine allocation problem. Virtual machine allocation, like other cloud computing technologies, is in a state of constant discovery. Literature [22] proposed an algorithm based on cloud agent, which aims to provide virtual machine allocation scheme for physical server. Literature [23] proposed an MMS virtual machine allocation algorithm based on min-max and shares features provided by the hypervisor. However, these algorithms do not take into account the uncertain factors of demand and the minimization conditions of price. A dynamic integration mechanism based on constraint programming is proposed in literature [24]. In literature [25], demand and price factors are considered, but response time and other issues are not taken into account. These algorithms are based on different application scenarios, and have their own characteristics. The existing algorithm, however, is mainly to study the internal allocation of resources on a single virtual machine. It is used to improve the utilization of vm physical resources and meet users' computing requirements by dividing physical computing resources [26]. In addition, most algorithms are put forward in specific demand scenarios, without considering various uncertainties in practical applications.

From the above analysis, we know that the above methods have studied virtual machine resource allocation optimization in cloud computing to some extent, but some problem still exists. On the other hand, no scholar has applied multiobjective genetic algorithm to this field till now, so the research here still draws a blank, which has great theoretical research and practical application value.

The contributions of this paper are as follows:

1. The proposed evolutionary algorithm provides a new solution idea for solving NP problems, and the emergence of multiobjective optimization algorithm brings a new solution idea for complex multiobjective optimization practical problems.
2. Because of its good development prospects, the multiobjective optimization problem has become a research hotspot in the academic world. In this paper, MOGANS- and MOEA/D-based multiobjective optimization scheduling schemes are proposed according to the solution idea of multiobjective optimization.

This paper consists of five parts. The first and second parts give the research status and background. The third part is the MOEA/D-based virtual machine resource allocation in cloud computing. The fourth part shows the experimental results and analysis. The experimental results of this paper are introduced, compared, and analyzed with relevant comparison algorithms followed. Finally, the fifth part summarizes the full paper.

## 3. MOEA/D-Based Virtual Machine Resource Allocation in Cloud Computing

### 3.1. Conventional Particle Swarm Optimization Algorithm

Conventional particle swarm optimization (PSO) is an intelligent bionic method, which was jointly proposed by social psychologists Kennedy and Dr. Eberhart in 1995. It has become the mainstream intelligent optimization processing method at present and has been comprehensively applied in the academic and industrial circles. Its optimization performance is mainly reflected in convergence [27, 28].

(1) $$\begin{array}{c} \chi =\frac{2}{\sqrt{{\left(2-l-\sqrt{l^{2}-4l}\right)}^{2}}},\;l=c_{1}+c_{2},l>4, \end{array}$$

where *χ* is the convergence factor of particle swarm. For the particle in each iteration, the particle changes its velocity and position according to the following formula:

(2) $$\begin{array}{c} v_{\text{id}}^{k+1}=v_{\text{id}}^{k}+\chi \left[c_{1}r_{1}\left(F_{\text{id}}-Q_{\text{id}}^{k}\right)+c_{1}r_{2}\left(H_{\text{gd}}-Q_{\text{id}}^{k}\right)\right], \end{array}$$

(3) $$\begin{array}{c} Q_{\text{id}}^{k+1}=Q_{\text{id}}^{k}+v_{\text{id}}^{k+1}, \end{array}$$

(4) $$\begin{array}{c} F_{\text{id}}⩾H_{\text{gd}}n, \end{array}$$

where *d*=*i*=1,2,…, *m*, *k* is the number of iterations, and *c*_1_, *c*_2_ are decay acceleration factors.

The (PSO) method here is determined by cloud user U and cloud VM, that is, the utility function of cloud user U is used to obtain the optimal position of particle search, and VM is used to obtain the optimal position of overall particle swarm search, that is, one particle represents one cloud user, The whole particle represents the virtual machine resources in the cloud computing environment of the whole field, which corresponds to the particle swarm optimization method. In this way, not only can each cloud user get the maximum benefit but also can make the whole cloud computing environment VM get global optimization.

At present, many scholars have put forward many excellent algorithms for solving multiobjective problems, among which the representative ones are NSGA-II, PSO, etc. However, these algorithms do not adopt decomposition thought in solving the MOP problem. These algorithms mainly deal with the MOP problem through some algorithms, and finally integrate the MOP into a whole. Then, it is solved by single objective optimization problem. But the downside of this is that it becomes very difficult to allocate fitness. However, many of the target values are independent, so there are many different cases of how to judge the superiority of a solution, and there are many other cases of the relationship between the solutions besides being dominant. Therefore, it is difficult to specify suitable fitness for these solutions to compare the advantages and disadvantages of these solutions in order to perform selection operations. In view of this problem, the decomposition idea is put forward, and the decomposition strategy idea is to transform multiple objectives into solving the problem of each single objective, mainly by solving the problem with multiple objectives. It is decomposed into a single solution optimization problem. By optimizing the solution of each sub-objective space, the solution of the whole solution set can be optimized to achieve the purpose of multiobjective optimization, and finally the global optimization solution based on Pareto is obtained. Therefore, MOEA/D comes into being.

### 3.2. Decomposition Strategy of MOEA/D Algorithm

The decomposition idea of MOEA/D is that, unlike other multiobjective optimization algorithms, the multiobjective problem is not processed as a whole through integration. Instead, it transforms the multiobjective solution into solving the single-objective optimization problem in *N* subspace through decomposition of the solution space. Among them, the solution in the subspace transforms a multiobjective problem into a single-objective problem by means of aggregation method, and the common aggregation methods include Chebyshev method, weighted sum method, and so on. In this paper, the aggregation function is implemented by Chebyshev:

(5) $$\begin{array}{c} \text{minimize }g^{\text{te}}\left(\frac{x}{y},z^{*}\right)=\underset{1\leq i\leq x}{\max}\left\{\lambda_{i}\left|f_{i}\left(x\right)-z_{i}^{*}\right|\right\},\\ \text{subject to}\;x\in \Omega n. \end{array}$$

And ∑_*i*=1_^*m*^*λ*_*i*_=1(∀*i*, *λ*_*i*_ ≥ 0), *z*_*∗*_=(*z*_1_^*∗*^, *z*_1_^*∗*^,…*z*_*m*_^*∗*^)^*T*^ are the reference points. And, *z*^*∗*^ is the optimal value of each target value in all solutions.

### 3.3. Implementation of MOGA-D Resource Optimization Scheme

The main characteristics of MOEA/D algorithm are as follows:

1. MOEA/D algorithm does not transform the multitarget values into a single-target progressive solution through the traditional method but decomposes the multitarget space, thus transforming the multitarget solution problem into the optimization problem of solving the sub-target space solution, and the sub-target space solution can be transformed into a single-target solution problem according to the vector of the solution space.
2. An excellent core idea of MOEA/D is to update the new solution by exchanging information of neighborhood solutions. This strategy greatly speeds up the search of understanding space, and has lower time and space complexity compared with some other multiobjective optimization solutions

Because the resource scheduling problem in cloud computing environment can finally be transformed into solving a multiobjective optimization problem through evolutionary algorithm, the resource scheduling scheme based on MOEA/D algorithm is proved by experiments to reduce the time complexity of the algorithm compared with MOGANS, so as to solve the shortage of MOGANS in computing performance.

The algorithm mainly consists of four parts: target space decomposition, population classification, crossover, and mutation. The core of the multiobjective optimization problem is to decompose the solution space into *N* sub-target space, where OMEGA represents the target space, so omega can be decomposed into a sub-target space composed of (Ω_1_, Ω_2_,…, Ω_*N*_). Individuals in the population can be mapped into different subspaces according to the generated sub-target space, so as to achieve the classification of the population. The vector composed of each target value of individuals in the initial population is mapped into N sub-target space to ensure that each sub-target space has a solution. The division of target space and population classification can be realized by the following formula:

(6) $$\begin{array}{c} p^{\text{i}}=\left\{x|x\in \text{POP},\Delta \left(F\left(x\right),\lambda^{i}\right)=\underset{1\leq j\leq N}{\max}\left\{\Delta \left(F\left(x\right),\lambda^{j}\right)\right\}\right\}, \end{array}$$

(7) $$\begin{array}{c} \Delta \left(F\left(x\right),\lambda^{i}\right)=\frac{\lambda^{i}*{\left(F\left(x\right)-Z\right)}^{T}}{\left\|\lambda^{i}\right\|*\left\|F\left(x\right)-Z\right\|},\;i=1,\ldots ,m, \end{array}$$

(8) $$\begin{array}{c} \Omega_{\text{i}}=\left\{F\left(x\right)|x\in \Omega ,\Delta \left(F\left(x\right),\lambda^{i}\right)=\underset{1\leq j\leq N}{\max}\left\{\Delta \left(F\left(x\right),\lambda^{j}\right)\right\}\right\}, \end{array}$$

where *Z* is a reference point and Δ(*F*(*x*), *λ*^*i*^) is the optimal solution of each target vector. It is also the cosine of the angle. In this way, POP population is divided into *N* classes. So there is a solution in every subspace and you have a variety of systems that help maintain the solution. Then, the multiobjective solving problem can be transformed into a single-objective optimization problem by the following aggregation function, which is in the form of the following:

(9) $$\begin{array}{c} \text{minimize }{\text{g}}^{te}\left(x|y,Z^{*}\right)=\underset{1\leq j\leq m}{\max}\left\{\frac{\left|f_{j}\left(x\right)-z_{j}^{*}\right|}{\lambda_{j}}\right\}. \end{array}$$

And the letters in formula (9) are the same as (6)–(8).

### 3.4. Optimization of VM Allocation Problems

In the optimization process, two schemes are proposed for users to choose. One is the scheme without considering the time factor, which only optimizes the cost and minimizes it while ignoring the time factor. It is suitable for users who do not care about time. Another kind is considering two factors of cost and time; the scheme firstly sets up a relatively small default time threshold. If the user has higher requirements, processing time can be dedicated to provide users with a time threshold that can be set up, after the cost optimization. The linear programming method is adopted, with the minimum cost as the optimization objective, and the processing time of resource price, virtual machine quantity, and supplier's supply capacity as the optimization conditions.

Formula (10) represents the optimization goal, namely, the overall cost; formula (11) indicates that the number of VMS obtained from the supplier in each usage phase does not exceed the number of VMS reserved. Equations (12)–(15) indicate that the total amount of basic resources obtained from each supplier should not exceed the upper limit of the maximum resources that the supplier can provide. Formula (15) indicates that the number of VMS in each stage of optimization is a nonnegative integer:

(10) $$\begin{array}{c} \text{Minimize}:\\ \sum_{p_{j}\in P}\sum_{v_{i}\in V}c_{j,i}^{\left[e\right]}X_{j,i}^{\left[e\right]}+\sum_{D\in \bar{D}}\sum_{p_{j}\in P}\sum_{v_{i}\in V}p\left(D\right)\left(c_{j,i}^{\left[u\right]}X_{j,i}^{\left[u\right]}\left(D\right)+c_{j,i}^{\left[a\right]}X_{j,i}^{\left[a\right]}\left(D\right)\right), \end{array}$$

(11) $$\begin{array}{c} \text{Subject to}:\\ X_{j,i}^{\left(u\right)}\left(D\right)\leq X_{j,i}^{\left(e\right)},v_{i}\in V,p_{i}\in P,D\in \bar{D}, \end{array}$$

(12) $$\begin{array}{c} \sum_{v_{i}\in V}n_{j,i}^{\left(a\right)}\left(X_{j,i}^{\left[u\right]}\left(D\right)+X_{j,i}^{\left[a\right]}\left(D\right)\right)\leq s_{j}^{\left(a\right)},\;D\in \bar{D}, \end{array}$$

(13) $$\begin{array}{c} \sum_{v_{i}\in V}n_{j,i}^{\left(b\right)}\left(X_{j,i}^{\left[u\right]}\left(D\right)+X_{j,i}^{\left[a\right]}\left(D\right)\right)\leq s_{j}^{\left(b\right)},\;D\in \bar{D}, \end{array}$$

(14) $$\begin{array}{c} \sum_{v_{i}\in V}n_{j,i}^{\left(c\right)}\left(X_{j,i}^{\left[u\right]}\left(D\right)+X_{j,i}^{\left[a\right]}\left(D\right)\right)\leq s_{j}^{\left(c\right)},\;D\in \bar{D}, \end{array}$$

(15) $$\begin{array}{c} X_{j,i}^{\left(e\right)},X_{j,i}^{\left[u\right]}\left(D\right),X_{j,i}^{\left[a\right]}\left(D\right)\in \left\{\text{int eger}\right\},\;v_{i}\in V,p_{i}\in P,D\in \bar{D}n. \end{array}$$

## 4. Experimental Results and Analysis

### 4.1. Introduction to Experimental Environment and Data Set

It includes a control node and four computing nodes [15] but due to limited hardware resources, this paper cannot provide a better cloud environment in the authentication algorithm, so a data center composed of 17 physical units is simulated. P1 serves as the controller node, and the other four compute nodes p2-P5 provide resources for users through virtualization [25]. As the algorithm in this paper optimizes the revenue of the cloud service provider and takes the maintenance cost of the cloud server into account in the virtual machine configuration stage, the cost quotation of the cloud server is set according to the pricing of Amazon EC2 virtual machine, as shown in Table 1.

### 4.2. Experimental Results Analysis

**Figure 3:**
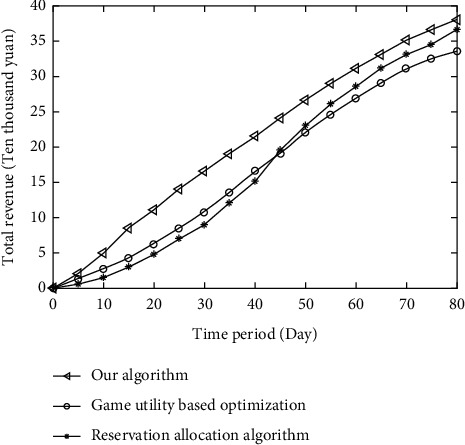
Total revenue comparison of cloud service providers.

Figure 3 shows the experimental results of the difference in total revenue between the algorithm in this chapter and the utility optimization algorithm based on non-cooperative game and resource reserve calculation method in cloud service providers. As the time period increases, the total revenue of single bit time is equal to that of the comparison algorithm.

The main reason is that the algorithm makes a reasonable planning of resources in the virtual machine configuration stage in this chapter, so as to maximize the revenue of cloud services and optimize resource allocation. However, the utility optimization algorithm based on cooperative game cannot guarantee the optimal game. With the increase of time cycle, both the algorithm in this article and the game-based utility optimization algorithm maintain good returns under the effect of utility function optimization, while the resource reservation allocation algorithm starts to allocate on-demand resources with higher prices.

**Figure 4:**
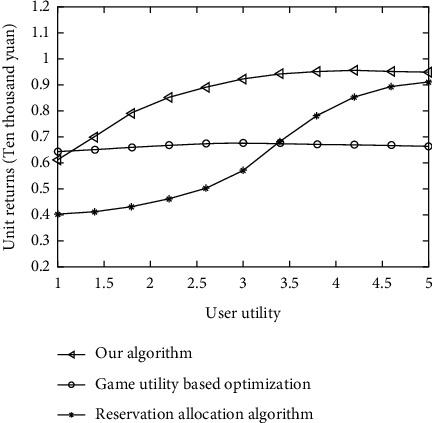
Comparison of unit revenue of different algorithms.

**Figure 5:**
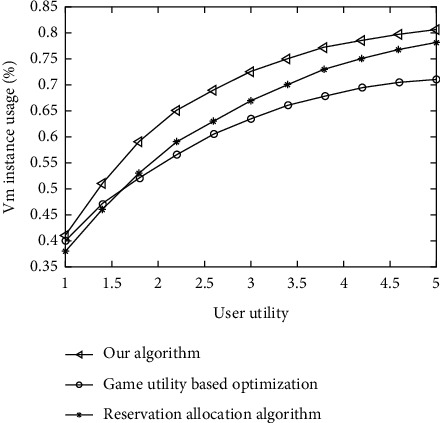
Comparison of VM instance usage.

Through the simulation experiment, the algorithm in this article is compared with the noncooperative game-based utility optimization algorithm, resource reservation algorithm, and user utility on the unit revenue of cloud data center. 0.4 is selected as the interval, and the statistical utility value is between [1, 5]. The experimental results are shown in Figures 4 and 5, with user utility on the horizontal axis and unit revenue on the vertical axis Revenue represents the revenue generated per request processed.

The experimental results show that with the increase of user utility, unit income also increases gradually, but the increasing trend gradually becomes slow, indicating that the utility function has a certain promoting effect on the increase of unit income, but this promoting effect will begin to fail after reaching a certain peak. At the stage of stimulative effect, the increase of yield per unit area is manifested. After reaching the peak, the stimulative failure is mainly related to the resource itself. Therefore, resources will not increase the unit infinite revenue of the non-cooperative game optimization algorithm with the increase of the utility function, the profit per unit increases steadily at first and then falls sharply, indicating that on-demand resources play a decisive role in the allocation process.

**Figure 6:**
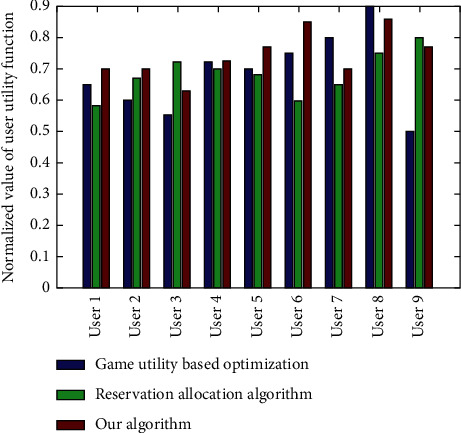
Comparison of utility values of different users under different algorithms.

In addition, the user utility difference between the algorithm in this article and the virtual machine instance based on noncooperative game utility optimization algorithm and resource reservation algorithm of the same user request is also compared as shown in Figure 6. The experimental results of actual satisfaction and maximum satisfaction ratio of users show that there is no significant difference between the proposed algorithm and other algorithms according to different users. Although the utility value differs greatly, the proposed algorithm is still effective on the whole. Combined with the analysis of the previous experimental results, it is proved that the algorithm in this article has higher revenue and resource utilization of cloud service providers and better overall performance without affecting user utility.

**Figure 7:**
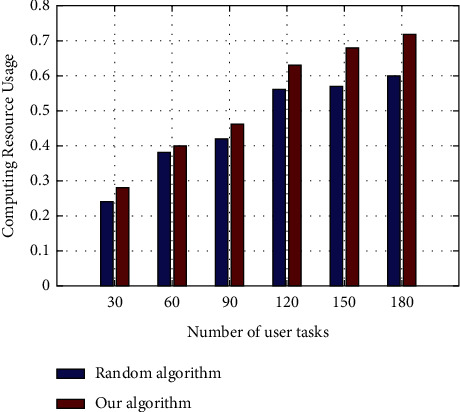
Comparison of computing resource usage.

The experiment compares the performance of OpenStack random scheduling algorithm and the algorithm in this article in computing resource CPU utilization, as shown in Figure 7. The horizontal axis represents the number of random user tasks, and the vertical axis represents the CPU resource utilization. This kind of performance results from the large amount of idle resources in the initial stage of resource allocation, which are used by users with the increase of user tasks. The reason is that the algorithm in this article classifies user tasks and physical machines, and computationally intensive users are in the utility function during resource allocation. In this way, computing resources can be fully utilized to improve computing resource utilization.

**Figure 8:**
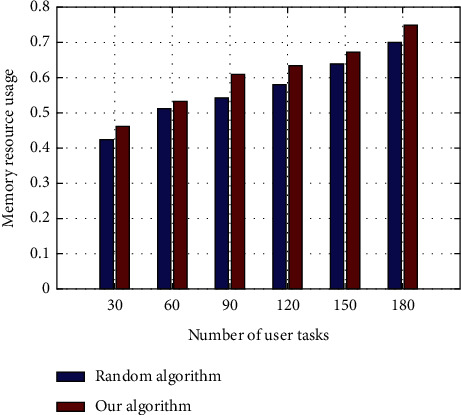
Comparison of memory resource usage.

The experimental results of memory resource utilization performance of OpenStack random scheduling algorithm and the algorithm in this article are compared as shown in Figure 8. The results in the Figure show that with the increase of the number of user tasks, resource utilization will increase steadily. The algorithm has a higher utilization of computing resources, but the difference between the two is not very large. The reason is that the host filtering in the Nova component of OpenStack gives priority to memory resource allocation and takes memory resource as the measurement standard. Therefore, the fluctuation of memory resource in the allocation process is smaller than that of the computing resource. Memory resource utilization has been improved to some extent.

Under the same initial conditions (the same initial state of VM distribution and the same load change of each application VM), the four algorithms are run to obtain the new distribution states of each VM, respectively. The number of migration times in the stable time and the number of idle physical nodes of these new distribution states are shown in Tables 2 and 3.

As can be seen in Tables 2 and 3, MOGANS makes a good balance between the stability of virtual machine distribution and the cost of virtual machine migration required by the old and new states, ensuring that the new multivirtual machine distribution state not only has a long stability time but also requires fewer virtual machine migration times from the current virtual machine distribution state to the new virtual machine distribution state. Although GA-N can change to a new VM distribution only after a minimum number of VM migrations, the new VM distribution can only maintain stability for a short time. Once the VM distribution becomes unstable, new dynamic resource configuration requests will be triggered, resulting in a new round of VM migration. Ga-NN takes green calculation into account, and the new virtual machine distribution state obtained uses the least active physical nodes, and the remaining number of free physical nodes is the largest, which is about 8 times the number of free physical nodes of MOGANS. Ga-NN takes green calculation into account, and the new virtual machine distribution state obtained uses the least active physical nodes, and the remaining number of free physical nodes is the largest, which is about 8 times the number of free physical nodes of MOGANS. This indicates that the new virtual machine distribution state obtained by GA-NN is more energy saving, but this energy saving state cannot be maintained for a long time. Once the distribution state becomes unstable, additional new dynamic resource allocation cost will be added, and the energy saving effect will be greatly reduced.

**Figure 9:**
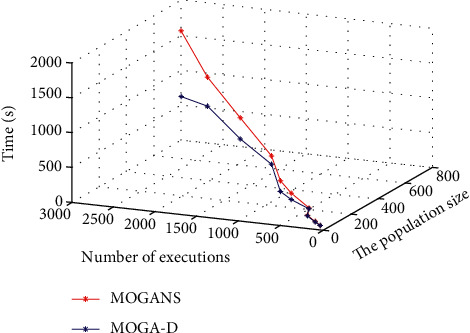
Comparison of running time cost between MOAGNS and MOGA-D.

In order to compare the computational overhead between MOGANS algorithm and MOGAD algorithm, the experimental data tested in this section are basically the same as the parameters as the former experiment, including two variables: population size and population total algebra. The values of population size and population total algebra are constantly changed to measure the running time of the algorithm, respectively. The experimental results are shown in Figure 9:

It can be seen from Figure 9 results that when the amount of data is small, the execution time cost of MOGANS and MOGA-D algorithm has little difference. When the amount of data increases sharply, the time cost of MOGA-D algorithm increases little. Therefore, when the amount of data is large, the calculation cost of MOGA-D algorithm is obviously superior as MOGANS has greatly improved the computational performance of the former.

## 5. Conclusions

Aiming at the deficiency of some current resource scheduling schemes of energy-saving resource scheduling in the current cloud computing environment, this paper proposes a multiobjective optimization scheme MOGANS based on NSGA-II multiobjective optimization.

However, MOGANS has insufficient computing performance, especially when the amount of data to be calculated increases, the performance of MOGANS is not satisfactory. After the test of the same amount of computation, the energy-saving resource scheduling scheme of the new opportunistic MOEA/D algorithm, MOGA-D, performs well. Compared with MOGANS, the computational performance is significantly improved with the same calculation scale. The similar multiobjective optimization results can also be obtained.

## Figures and Tables

**Table 1:** Cloud data center parameters.

Node	CPU (MIPS)	CPU cores	Memory (GB)	Bandwidth (M)
P1	3000	2	4	100
P2	2000	1	2	100
P3	1800	2	1	100
P4	2700	1	1	100
P5	2300	2	2	100

**Table 2:** Algorithms' performance (16 physical nodes, 42 virtual machines).

Algorithms	Number of migration	Stable time	Number of idle physical nodes
MOGANS	21	15	1.6
GA-S	6.8	3.8	0.5
GA-N	9.2	1.2	3.7
GA-NN	22.5	0.9	5.6

**Table 3:** Algorithms' performance (32 physical nodes, 84 virtual machines).

Algorithms	Number of migration	Stable time	Number of idle physical nodes
MOGANS	68	15.2	1.4
GA-S	81.5	13.8	0.9
GA-N	19.2	1.2	5.7
GA-NN	72.5	0.8	15.6

## Data Availability

The data used to support the findings of this study are available from the corresponding author upon request.
